# Eco-Friendly Microwave Synthesis of Sodium Alginate-Chitosan Hydrogels for Effective Curcumin Delivery and Controlled Release

**DOI:** 10.3390/gels10100637

**Published:** 2024-10-02

**Authors:** Ivan Ristić, Ljubiša Nikolić, Suzana Cakić, Vesna Nikolić, Jelena Tanasić, Jelena Zvezdanović, Marija Krstić

**Affiliations:** 1 Faculty of Technology Novi Sad, University of Novi Sad, Bulevar cara Lazara 1, 21000 Novi Sad, Serbia; jelenatanasic@uns.ac.rs; 2 Faculty of Technology, University of Niš, Bulevar Oslobodjenja 124, 16000 Leskovac, Serbia; nljubisa@tf.ni.ac.rs (L.N.); cakics@tf.ni.ac.rs (S.C.); nikolicvesna@tf.ni.ac.rs (V.N.); jzvezdanovic@tf.ni.ac.rs (J.Z.)

**Keywords:** alginate-chitosan hydrogels, microwave synthesis, curcumin bioavailability, green chemistry, controlled drug delivery

## Abstract

In this study, we developed sodium alginate-chitosan hydrogels using a microwave-assisted synthesis method, aligning with green chemistry principles for enhanced sustainability. This eco-friendly approach minimizes chemical use and waste while boosting efficiency. A curcumin:2-hydroxypropyl-β-cyclodextrin complex was incorporated into the hydrogels, significantly increasing the solubility and bioavailability of curcumin. Fourier Transform Infrared Spectroscopy (FTIR) analysis confirmed the structure and successful incorporation of curcumin, in both its pure and complexed forms, into the polymer matrix. Differential scanning calorimetry revealed distinct thermal transitions influenced by the hydrogel composition and physical cross-linking. Hydrogels with higher alginate content had higher swelling ratios (338%), while those with more chitosan showed the lowest swelling ratios (254%). Scanning Electron Microscopy (SEM) micrographs showed a porous structure as well as successful incorporation of curcumin or its complex. Curcumin release studies indicated varying releasing rates between its pure and complexed forms. The chitosan-dominant hydrogel exhibited the slowest release rate of pure curcumin, while the alginate-dominant hydrogel exhibited the fastest. Conversely, for curcumin from the inclusion complex, a higher chitosan proportion led to the fastest release rate, while a higher alginate proportion resulted in the slowest. This study demonstrates that the form of curcumin incorporation and gel matrix composition critically influence the release profile. Our findings offer valuable insights for designing effective curcumin delivery systems, representing a significant advancement in biodegradable and sustainable drug delivery technologies.

## 1. Introduction

In recent years, the field of pharmacokinetics and drug delivery has increasingly focused on developing innovative technologies to address the challenges of low aqueous solubility and suboptimal distribution pathways of numerous bioactive compounds. Polymeric materials, known for their advantageous physicochemical and biological properties, have shown great potential in addressing these challenges by ensuring that an optimal drug concentration is delivered to the target site for a sufficient duration to achieve the desired therapeutic response [1,2]. Consequently, there is significant interest in the development of polymeric formulations as drug delivery systems with diverse architectures, including micelles [3], membranes [4], nanoparticles [5], films [6], dendrimers [7], and hydrogels [8]. Hydrogels, in particular, have been extensively utilized as effective tools for targeted drug delivery due to their ability to increase drug solubility and reduce side effects. These systems can be precisely engineered to respond to environmental or external stimuli, thereby facilitating controlled and targeted drug release [9].

Natural polymers have attracted significant attention in recent drug delivery formulation research due to their superior biocompatibility and biodegradability. Among these, alginate and chitosan are particularly promising and have been extensively utilized in the pharmaceutical industry for controlled drug delivery applications [10,11]. Alginate (Alg) is an anionic, marine-derived linear polysaccharide, while chitosan (Chi) is a cationic polysaccharide obtained from the N-deacetylation of chitin. The presence of amino groups in chitosan and carboxyl groups in alginate renders these materials pH-sensitive, enhancing their utility as drug carriers. The individual use of chitosan or alginate is often associated with suboptimal mechanical properties and degradation profiles. However, their combination can significantly improve stability under acidic conditions, preventing the rapid diffusion of encapsulated drugs at low pH levels, as well as reducing enzymatic degradation and hydrolysis [12,13]. Nalini et al. [14] successfully encapsulated the bioflavonoid quercetin in synthesized and optimized Alg-Chi nanoparticles prepared by the ionic gelation method. Similarly, Sohail et al. [15] demonstrated that Alg-Chi nanoparticles, designed with both anionic and cationic outer layers, can be used as an effective drug delivery system for sustained and controlled amygdalin release, enhancing its cytotoxic effect on cancerous cells while protecting normal cells and tissues. Moreover, the Alg-Chi system has been investigated for the delivery of drugs such as insulin [16] and hesperidin [17], with a particular focus on the delivery of liposoluble components, including curcumin, in current research.

Curcumin, a natural compound derived from the plant species *Curcuma longa*, is well-known for its excellent antimicrobial, antiviral, antioxidant, and anti-inflammatory properties [18,19]. However, its broader therapeutic use is hindered by its poor solubility in water. To address this issue, numerous studies have focused on developing effective formulations to enhance its bioavailability. Formulations of curcumin with hydrogels, micelles, or cyclodextrins have been recognized for improving its physical and chemical properties. Recent research indicates that complexation with cyclodextrins is an effective strategy for solubilizing hydrophobic compounds, thereby enhancing the encapsulation process of bioactive compounds. Nikolić et al. [20] introduced a formulation of curcumin complexed with 2-hydroxypropyl-β-cyclodextrin in a thermosensitive hydrogel based on N-isopropylmethacrylamide and N-isopropylacrylamide. This formulation facilitated the incorporation of curcumin into the hydrogel by increasing its solubility. Zhao et al. [21] synthesized a biocompatible and biodegradable β-glycerophosphate/chitosan hydrogel system with an encapsulated curcumin:β-cyclodextrin complex to enhance the wound healing process.

Several studies have aimed to enhance the bioavailability and targeted delivery of curcumin using an alginate-chitosan drug delivery system. Silva et al. [22] encapsulated curcumin in Alg-Chi beads using deep eutectic solvents (DES) as drug solubilizing vehicles. Hydrogel beads were successfully produced using the extrusion-dripping method. Abbasalizadeh and colleagues [23] concluded that curcumin-chrysin-loaded alginate-chitosan hydrogels significantly reduced viability and induced apoptosis in breast and lung cancer cell lines, indicating their potential as an effective anticancer drug delivery system due to their enhanced bioavailability and stability. However, much of the existing research has focused on chemically cross-linked Alg-Chi hydrogels. The use of chemical cross-linkers necessitates an additional removal step, which is sometimes incomplete and can cause significant problems in biomedical applications [24]. Therefore, physical cross-linking using microwaves presents a more efficient alternative by promoting additional bonds through hydrogen bonding and/or electrostatic interactions without the need for cross-linker removal, thus eliminating the risk of hydrogel contamination. Albarqi et al. [25] reported on chitosan and sodium alginate hydrogels loaded with curcumin and synthesized with microwave assistance. Their findings indicated that microwave treatment significantly enhanced the swelling capacity, reduced erosion, and improved surface texture and drug content compared to untreated samples. These hydrogel membranes demonstrated wound healing potential with the controlled release of the incorporated drug. However, their synthesis required drying in an oven for 72 h. To the best of our knowledge, no data have been reported on the synthesis of Alg-Chi hydrogels achieved entirely in a single step using microwaves. This represents the originality and novelty of our work, as we have developed a method for one-step microwave synthesis of fully biodegradable sodium alginate-chitosan hydrogels, ensuring biocompatibility without the need for extensive post-synthesis treatment.

The aim of this work is to address two significant challenges in the field of targeted drug delivery. The first objective is to minimize the use of organic solvents or cross-linking agents that could compromise the biocompatibility of the drug delivery system. The second objective is to overcome the difficulties associated with encapsulating lipophilic compounds, such as curcumin, in polysaccharide hydrogels. To achieve these objectives, we developed a novel method for synthesizing fully biodegradable, multifunctional sodium alginate-chitosan hydrogels using microwaves. This method reduces the consumption of chemicals, energy, and time, and eliminates waste entirely. In this study, we incorporated a complex of curcumin with 2-hydroxypropyl-β-cyclodextrin into the pH-sensitive alginate-chitosan hydrogels to enhance the solubility of curcumin. This system aims to provide both targeted delivery and controlled release of curcumin, thereby contributing to the advancement of biocompatible and efficient drug delivery technologies.

## 2. Results and Discussion

### 2.1. FTIR Results

**Figure 1:**
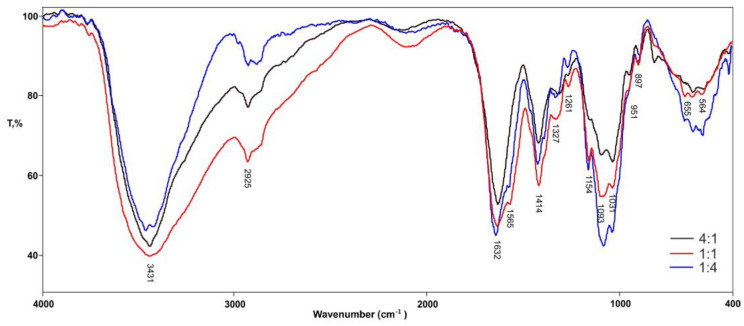
FTIR spectra of hydrogels 4A_1CH; 1A_1CH; and 1A_4CH.

The FTIR analysis of the physically cross-linked Alg-Chi hydrogels with varying ratios (1A_1CH, 1A_4CH, and 4A_1CH) provides valuable insights into the successful incorporation of both polymers and the intermolecular interactions responsible for hydrogel network formation (Figure 1). The consistent presence of a broad peak around 3420 cm^−1^ across all hydrogels, attributed to extensive hydrogen bonding (O–H stretching), underscores the critical role of these interactions in physical cross-linking and hydrogel stability. The intensity variations of this peak, particularly its prominence in the chitosan-rich (1:4) hydrogel, suggest the influence of polymer composition on the extent of hydrogen bonding within the network.

The presence of characteristic peaks for both alginate and chitosan further confirms their successful incorporation. The amide I and amide II vibrations at 1630–1636 cm^−1^ and 1565 cm^−1^, respectively, are indicative of chitosan, with higher intensity observed in the chitosan-rich hydrogel, aligning with its increased chitosan content [26,27].

Conversely, the alginate-rich (4:1) hydrogel displays a prominent peak at 1417 cm^−1^, corresponding to the asymmetric and symmetric stretching vibrations of carboxylate groups, a hallmark of alginate’s presence. The fingerprint region (1260–895 cm^−1^) further corroborates the presence of both polymers in all hydrogels, with subtle variations depending on the ratio, reflecting the influence of polymer composition on the overall spectral fingerprint. These findings are consistent with previous studies on Alg-Chi hydrogels, which have demonstrated the successful formation of physically cross-linked networks through intermolecular interactions, primarily hydrogen bonding, between the two polymers [28,29].

The FTIR analysis not only confirms the successful fabrication of the hydrogels but also highlights the impact of the polymer ratio on the network structure and intermolecular interactions, which can ultimately influence the hydrogels’ properties and performance.

**Figure 2:**
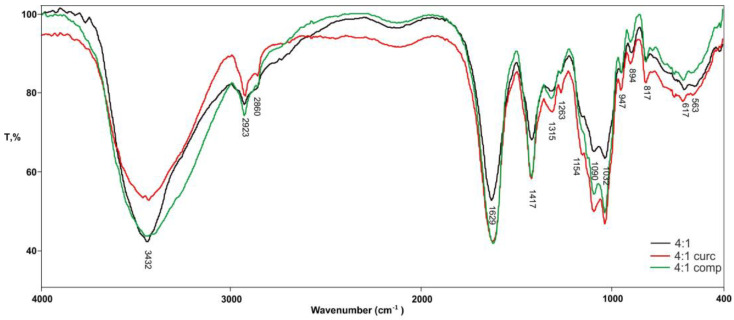
FTIR spectra of pure hydrogels 4A_1CH; with incorporated curcumin; and complex.

Figure 2 shows the comparative FTIR spectra of pure hydrogel 4A_1CH and hydrogels in which curcumin and its complex are incorporated.

The FTIR analysis of the 4A_1CH hydrogel, both with and without curcumin, provides valuable insights into the successful incorporation and interaction of curcumin within the hydrogel matrix. The data allow for a comparative analysis of the incorporation of curcumin and the curcumin-2-hydroxypropyl-β-cyclodextrin complex into the hydrogel. The FTIR spectrum of pure curcumin exhibits characteristic peaks at 3500 cm^−1^ (O–H stretching), 1628 cm^−1^ (C=O stretching), and 1510 cm^−1^ (C=C stretching) [20], which serve as reference points for identifying curcumin’s presence and interactions within the hydrogel. The 4A_1CH hydrogel, without curcumin, displays a broad peak at 3432 cm^−1^ (O–H stretching) and strong peaks at 1629 cm^−1^ and 1417 cm^−1^, corresponding to carboxylate groups in alginate. The incorporation of curcumin into the hydrogel results in the appearance of curcumin-specific peaks, albeit with slight shifts and intensity changes, indicating interactions with the hydrogel matrix. The O–H peak broadens (3427 cm^−1^), suggesting hydrogen bonding between curcumin and the hydrogel. Peaks at 1619 cm^−1^ and 1507 cm^−1^ confirm the presence of curcumin’s C=O and C=C bonds, respectively. The spectrum of the hydrogel with complexed curcumin shows more pronounced shifts and changes in intensity compared to the hydrogel with pure curcumin. The broader O–H peak (3436 cm^−1^) and the shift in the C=O peak (1618 cm^−1^) indicate stronger interactions between the complexed curcumin and the hydrogel matrix, likely through enhanced hydrogen bonding. This suggests that the curcumin-2-hydroxypropyl-β-cyclodextrin complex is better incorporated into the hydrogel than curcumin alone. The enhanced interaction is likely due to the ability of cyclodextrin to form inclusion complexes with curcumin, improving its solubility and facilitating its interaction with the hydrogel network [30,31].

**Figure 3:**
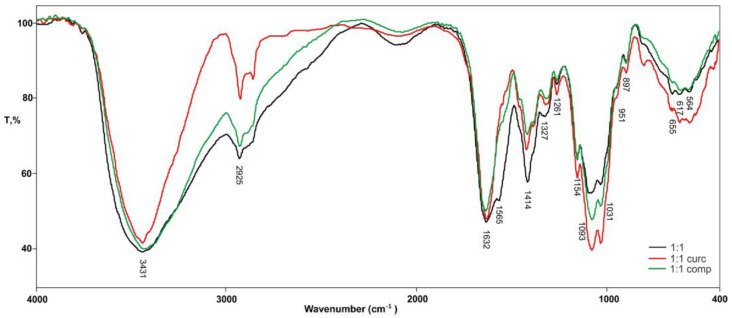
FTIR spectra of pure hydrogels 1A_1CH; with incorporated curcumin; and with complex.

Figure 3 shows the comparative FTIR spectrum of pure hydrogel 1A_1CH and hydrogels in which curcumin and its complex are incorporated.

FTIR analysis of the 1A_1CH hydrogels loaded with either curcumin or a curcumin-2-hydroxypropyl-β-cyclodextrin (HPβCD) complex reveals the successful incorporation of both forms of curcumin within the hydrogel matrix. The characteristic C=O stretching peak of curcumin, observed around 1625 cm in both hydrogels, confirms its presence. The broadening of the O–H peak (around 3433 cm^−1^) in both curcumin-loaded hydrogels suggests the formation of hydrogen bonds between curcumin and the hydrogel matrix. This interaction is further supported by the slight shift in the C=O peak (from 1628 cm^−1^ in pure curcumin to 1629 cm^−1^ and 1633 cm^−1^ in the hydrogels with curcumin and the complex, respectively).

**Figure 4:**
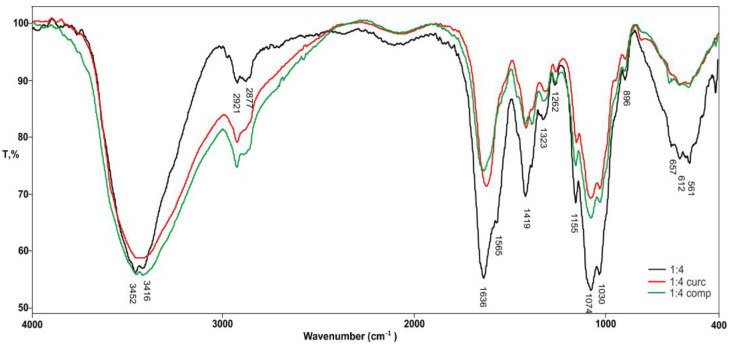
FTIR spectra of pure hydrogels 1A_4CH; with incorporated curcumin; and with complex.

Figure 4 shows the comparative FTIR spectrum of pure hydrogel 1A_4CH and hydrogels in which curcumin and its complex are incorporated.

The FTIR spectra of (1A_4CH) hydrogels loaded with either curcumin or a curcumin-2-hydroxypropyl-β-cyclodextrin (HPβCD) complex reveal successful incorporation of both forms within the hydrogel matrix. Characteristic peaks of curcumin (1627 cm^−1^: C=O stretching, 1508 cm^−1^: C=C stretching) and the complex (1027 cm^−1^: C–O–C stretching, 1155 cm^−1^: C–OH stretching) confirm their presence. Notably, the higher intensity of the curcumin-specific peaks in the hydrogel loaded with pure curcumin suggests a potentially higher degree of incorporation or stronger interactions with the hydrogel matrix compared to the complexed form. This observation could be attributed to the greater accessibility of curcumin’s functional groups for interaction with the hydrogel components in the absence of cyclodextrin.

The relative FTIR peak intensities suggest a higher incorporation or stronger interaction of curcumin in the 1A_4CH hydrogel, as well as a higher incorporation of the curcumin-HPβCD complex in the 4A_1CH hydrogel. This indicates that the alginate:chitosan ratio significantly influences the incorporation efficiency of both curcumin and its complex, highlighting the importance of tailoring hydrogel composition for optimal drug loading in specific applications.

### 2.2. DSC Results

**Figure 5:**
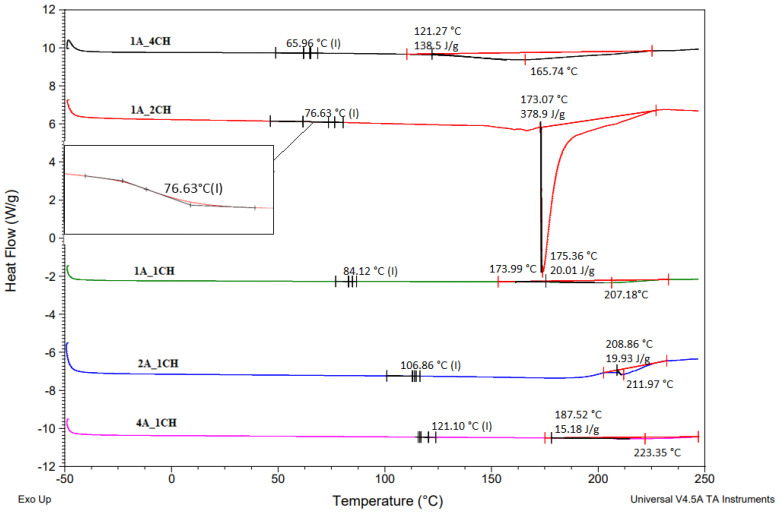
DSC thermographs of Alg-Chi hydrogels.

Differential scanning calorimetry tests performed for hydrogels 4A_1CH; 2A_1CH; 1A_1CH; 1A_2CH; and 1A_4CH are presented in Figure 5.

Differential scanning calorimetry (DSC) analysis of the alginate-chitosan hydrogels revealed distinct thermal transitions influenced by the composition and physical cross-linking within the hydrogel networks. Figure 5 shows the DSC curves of the samples, where the glass transition temperature (Tg) is indicated by the symbol (I). The increase in Tg with higher chitosan content is attributed to chitosan’s rigidity and extensive hydrogen bonding, which limit the mobility of polymer chains. The specific T_g_ values obtained from the DSC curves were 69.19 °C (1A_4CH), 75.74 °C (1A_2CH), 84.21 °C (1A_1CH), 106.46 °C (2A_1CH), and 125.92 °C (4A_1CH). These results indicated the partial miscibility of the polysaccharides used [28]. Moreover, studies have reported that physical cross-linking, such as hydrogen bonding and ionic interactions between alginate and chitosan, can significantly influence T_g_ and T_m_ values in these hydrogels [32].

The melting temperature (T_m_) was influenced by both the alginate:chitosan ratio and the extent of cross-linking. The crystalline domains in the hydrogels likely originated from the alginate component, as chitosan is generally amorphous. However, chitosan’s presence and interactions with alginate can disrupt the crystalline structure, leading to variations in T_m_. Higher chitosan content might decrease T_m_ due to the disruption of alginate’s crystalline domains. The T_m_ values (and corresponding enthalpies of melting, ΔHm) for the different hydrogel formulations were 122.87 °C (130.8 J/g) for 1A_4CH, 173.06 °C (379.1 J/g) for 1A_2CH, 178.55 °C (16.15 J/g) and 207.25 °C for 1A_1CH, 208.90 °C (16.44 J/g) and 211.92 °C for 2A_1CH, and 187.50 °C (13.31 J/g) and 226.22 °C for 4A_1CH. The presence of multiple melting peaks in some samples suggests the existence of different types of crystalline domains or varying degrees of crystallinity within the hydrogel networks. At temperatures higher than 250 °C, there is a shift in the baseline and an indication of the appearance of an exothermic peak that may be related to the decomposition of amine (GlcN) units at 295 °C [33].

The interplay between the alginate-chitosan ratio, the nature of cross-linking, and the resulting chain mobility and crystallinity are crucial factors for determining the thermal behavior of these hydrogels. Understanding these relationships is crucial for tailoring the properties of alginate-chitosan hydrogels for specific applications [34].

**Figure 6:**
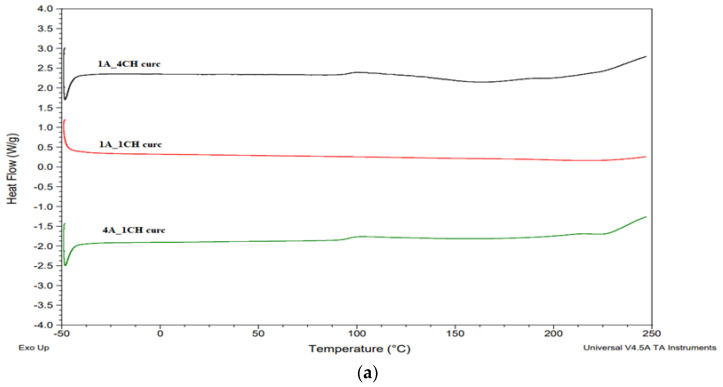

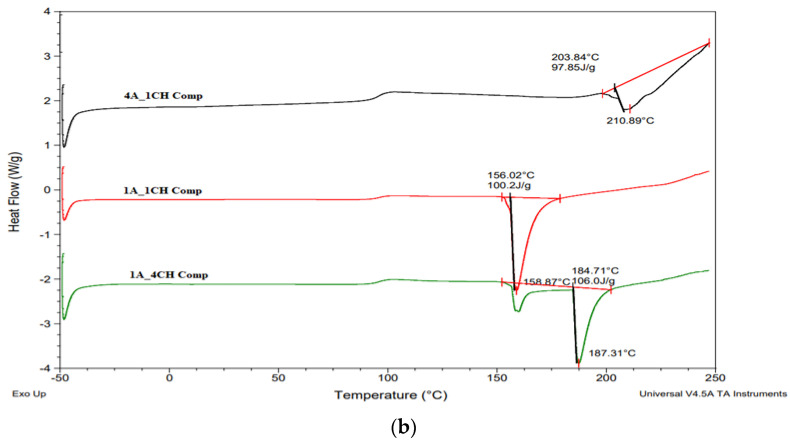
DSC thermographs of Alg-Chi hydrogels with incorporated (**a**) curcumin and (**b**) curcumin-2-hydroxypropyl-β-cyclodextrin complex.

DSC analysis of curcumin-incorporated hydrogel samples (Figure 6a) demonstrates consistent thermal profiles compared to pure hydrogel controls. The absence of a distinct endothermic peak at 187 °C, characteristic of crystalline curcumin [20], suggests the desired dispersion or amorphous state of curcumin within the hydrogel matrix rather than surface deposition or crystalline aggregation. This amorphous state may enhance the solubility and bioavailability of curcumin, potentially facilitating sustained release and therapeutic efficacy from the hydrogel formulation.

DSC analysis of complex-incorporated hydrogel samples reveals differential integration patterns and associated thermal properties (Figure 6b). The absence of the characteristic endothermic peak of curcumin at 187 °C in samples 1A_1CH_Com and 4A_1CH_Com suggests complete molecular dispersion or complexation of the curcumin complex within the hydrogel matrix. Conversely, the presence of this peak in sample 1A_4CH_Com indicates incomplete integration, potentially due to partial surface deposition or aggregation of the complex.

Furthermore, a decrease in the melting temperature of all three samples compared to pure hydrogels is observed. This is attributed to the incorporation of the curcumin complex, which possesses a lower melting point than the hydrogel matrix. This phenomenon suggests intermolecular interactions between the complex and the hydrogel network, potentially influencing the thermal stability, phase behavior, and ultimately the drug release kinetics and bioavailability of these hydrogel formulations.

These findings underscore the complex interplay between curcumin complex incorporation mechanisms, resulting thermal properties, and potential implications for the controlled release and therapeutic efficacy of curcumin from these hydrogel systems.

### 2.3. Swelling Properties

**Figure 7:**
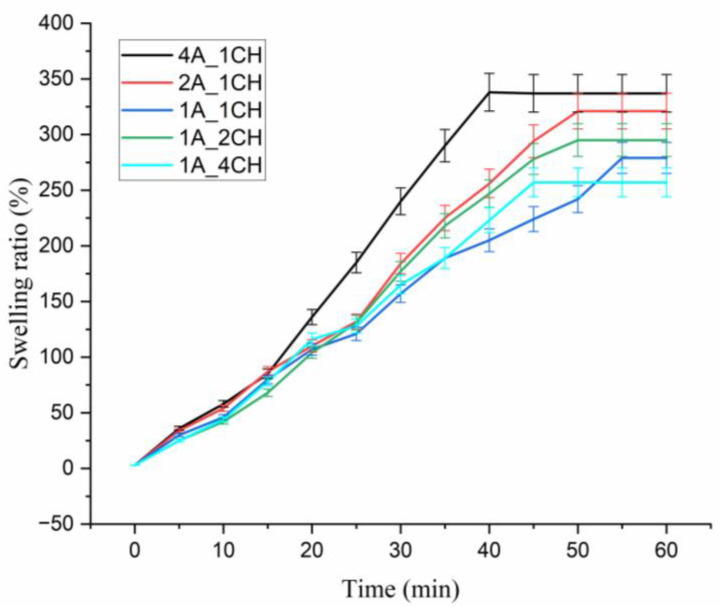
Swelling of Alg-Chi hydrogels at pH 7.4.

The swelling testing was done for 60 min at room conditions in solutions with pH values of 3.4, 6.7, and 7.4. The results for swelling at room conditions in a neutral medium are shown in Figure 7.

The swelling behavior of hydrogels synthesized via microwave irradiation was investigated, revealing the significant influence of the alginate:chitosan ratio on hydrogel water absorption capacity. Hydrogels with a higher proportion of alginate (4A_1CH, 2A_1CH) demonstrated significantly higher swelling ratios, reaching equilibrium of approximately 350% after 40 min. This is attributed to alginate’s hydrophilic nature, which promotes hydrogen bonding with water molecules, and its ability to form expansive gel networks. Conversely, hydrogels with a higher proportion of chitosan (1A_2CH, 1A_4CH) exhibited a lower swelling ratio, equilibrating at around 250% after 40 min. This reduction is due to increased cross-linking density resulting from interactions between the amino groups of chitosan and the carboxyl groups of alginate, forming a denser network that restricts water absorption. The hydrogel with an equal proportion of alginate and chitosan (1A_1CH) displayed an intermediate swelling ratio, equilibrating at around 270%, suggesting a balance between the hydrophilic nature of alginate and the cross-linking effect of chitosan. These findings highlight the importance of the composition and physical cross-linking in modulating hydrogel swelling behavior. The non-covalent interactions between alginate and chitosan create a network structure that resists excessive swelling. Understanding the interplay between polymer composition and cross-linking density is crucial for tailoring the swelling properties of hydrogels for specific applications, such as drug delivery or tissue engineering [35].

Quantitative analysis of hydrogel swelling behavior across a range of media with varying pH values demonstrates minimal influence of the pH value on the degree of swelling; see Table 1. While minor deviations in swelling degree are observed in samples with one excess component, these variations are statistically insignificant and remain within the expected range for the intended physiological release environment of the medical substance. This suggests a robust and consistent swelling response of the hydrogel across a range of physiological conditions, potentially contributing to predictable and controlled drug release kinetics.

### 2.4. SEM

**Figure 8:**
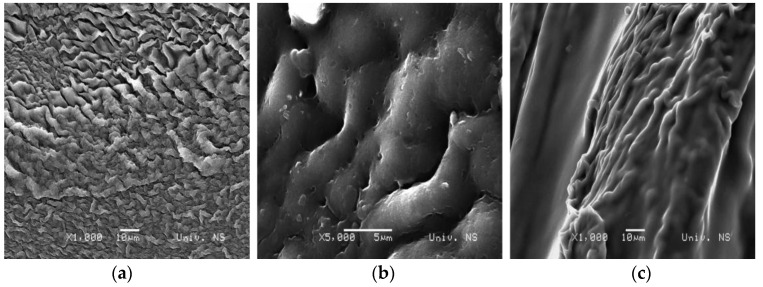
SEM micrographs of samples: (**a**) 1A_1CH; (**b**) 1A_4CH; (**c**) 4A_1CH.

SEM micrographs of Alg:Chi xerogels with the ratio 1:1, 1:4, and 4:1, respectively, are shown in Figure 8. Samples 1A_1CH and 1A_4CH (Figure 8a,b) have a porous structure, where sample 1A_1CH has smaller pores, with the size 100 + 20 nm, while sample 1A_4CH has pores with the size 300–400 nm. Sample 1A_4CH also has micropores 1.5–3 μm in size. In the morphology of sample 4A_1CH in the form of xerogel, no pores can be observed, but rather large folds. As the gels are dry, the polymer networks can collapse, leaving a rough surface [36].

**Figure 9:**
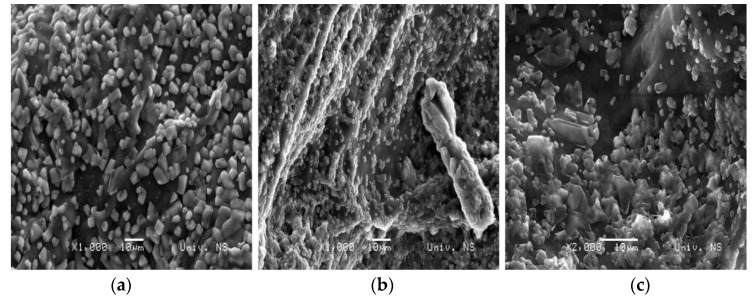
SEM micrographs of the xerogels with incorporated curcumin: (**a**) 1A_1CH_Cu; (**b**) 1A_4CH_Cu; (**c**) 4A_1CH_Cu.

The SEM micrographs presented in Figure 9 illustrate the surfaces of gel samples with incorporated pure curcumin. In the 1A_1CH_Cu and 1A_4CH_Cu samples, the surface is uniformly covered with curcumin particles, and the pores, which are visible in the SEM micrographs of the pure xerogels (Figure 8), are covered, indicating that curcumin is embedded within these pores. In contrast, the 4A_1CH_Cu sample exhibits a less uniform distribution of curcumin on the surface, with a lower overall presence of curcumin.

**Figure 10:**
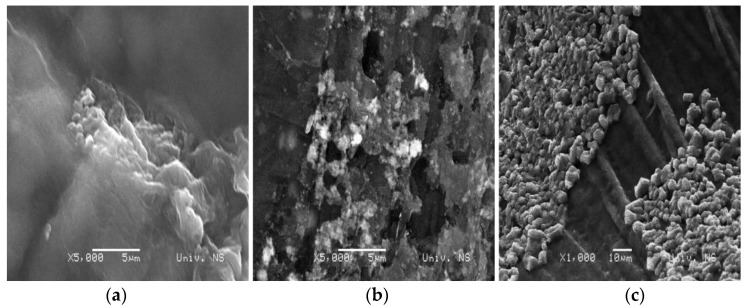
SEM micrographs of the xerogels with incorporated complex curcumin:2-hydroxypropyl-β-cyclodextrin: (**a**) 1A_1CH_Com; (**b**) 1A_4CH_Com; (**c**) 4A_1CH_Com.

Figure 10 presents SEM micrographs of xerogels with incorporated complex curcumin:2-hydroxypropyl-β-cyclodextrin. The inclusion of the complex did not affect the morphology of the gels, and the images shown confirm its successful incorporation into the gels, which is in accordance with the data from the FTIR analysis.

### 2.5. Loading Efficiency and Release of Curcumin from Alg-Chi Hydrogels

The curcumin incorporation efficiency, η, results are presented in Table 2. Despite curcumin’s insolubility in water, the incorporation efficiency of pure curcumin ranges from approximately 30% to 50% affected by the hydrogels’ composition. This relatively good incorporation for the insoluble substance is probably due to the high degree of swelling of the hydrogels themselves and the chemical structure of used reactive components. However, these incorporation efficiencies are suboptimal. To enhance curcumin incorporation, we utilized a curcumin:2-hydroxypropyl-β-cyclodextrin complex, which significantly improved the incorporation rates into the hydrogel samples. The incorporation efficiency of curcumin with the inclusion complex was highest for the 1A_1CH_Com sample, reaching 87.7%, and lowest for the 1A_4CH_Com sample, at 76.7%, aligning with the observed swelling behavior of the synthesized gels. The complexation with 2-hydroxypropyl-β-cyclodextrin increases curcumin’s solubility in water by 1237-fold [20], facilitating its incorporation into the synthesized hydrogels. Kasapoglu-Calik and Ozdemir [37] investigated the incorporation of the curcumin-β-cyclodextrin inclusion complex into poly(N-isopropylacrylamide/Na alginate) hydrogels, achieving a maximum incorporation efficiency of under 80%. This comparison supports our hypothesis that the alginate-chitosan hydrogel system synthesized as described in this work can significantly enhance the solubility and bioavailability of curcumin.

**Figure 11:**
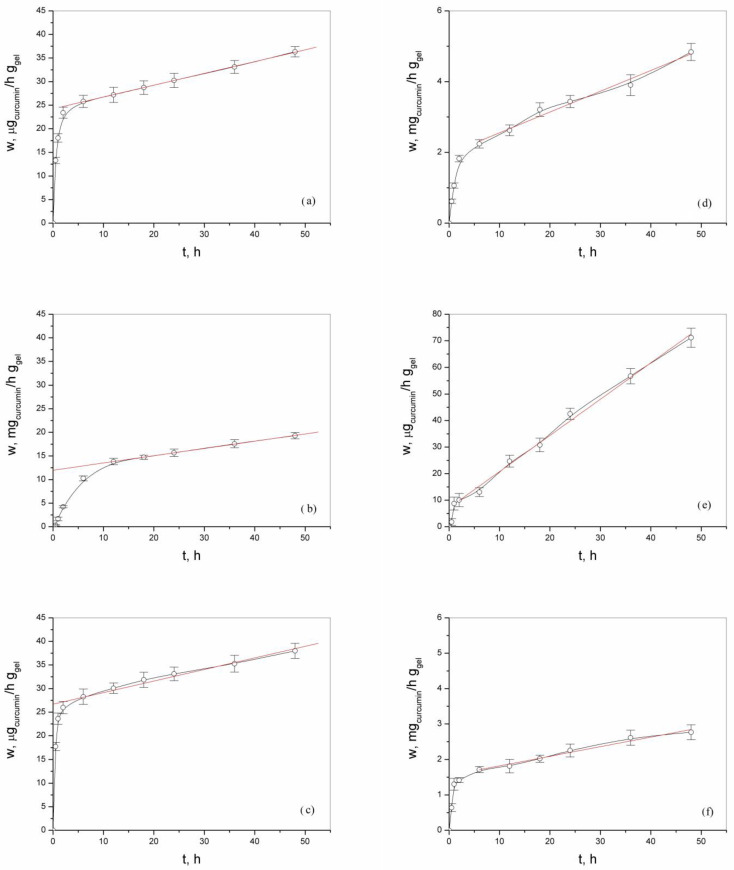
Profile of curcumin release, depending on time (t) in hours (h), from: (**a**) 1A_4CH_Cu, (**b**) 1A_4CH_Com, (**c**) 1A_1CH_Cu, (**d**) 1A_1CH_Com, (**e**) 4A_1CH_Cu, and (**f**) 4A_1CH_Com.

The curcumin release study evaluated the release rates of curcumin incorporated in two forms—pure and inclusion complex of curcumin:2-hydroxypropyl-β-cyclodextrin—from alginate-chitosan gels. The results are summarized in Table 2. The release profiles of curcumin from alginate-chitosan hydrogels are shown in Figure 11. In these figures, it can also be seen that there is an initial release of a significant amount of curcumin in the first 2 h of monitoring, whereas in samples with an incorporated complex, the release is more consistent and uniform over time.

The release profiles of curcumin from physically cross-linked alginate-chitosan hydrogels were analyzed using the following equations:

- 1A_4CH_Cu: w = 24.236 + 0.25 × t (Release rate: 0.25 µg/h/g of gel)
- 1A_1CH_Cu: w = 26.681 + 0.245 × t (Release rate: 0.245 µg/h/g of gel)
- 4A_1CH_Cu: w = 7.132 + 1.363 × t (Release rate: 1.363 µg/h/g of gel)

The results demonstrate the clear dependence of curcumin release rate on the alginate:chitosan ratio. The chitosan-dominant hydrogel (1:4) exhibited the slowest release rate, while the alginate-dominant hydrogel (4:1) exhibited the fastest. This suggests that the interaction between alginate and chitosan plays a crucial role in controlling drug release. A higher proportion of chitosan likely leads to a denser network structure, hindering curcumin diffusion and resulting in slower release. When curcumin is incorporated in its pure form, the release rate is highest for the 4A_1CH_Cu sample (1363 µg/h g_gel_), while for the 1A_4CH_Cu and 1A_1CH_Cu samples, that value was more than 5 times lower (0.25 µg/h g_gel_ and 0.245 µg/h g_gel_, respectively). This observation could be due to the weaker nucleophilic behavior of alginate, which is oxygen-rich, compared to chitosan, which is nitrogen-rich [2]. This suggests a stronger interaction between curcumin and chitosan, which is in accordance with FTIR results, resulting in a slower release rate of curcumin from the gels with a higher amount of chitosan. These results are supported by SEM micrographs of these samples, which show that the surface of the xerogels with a higher ratio of chitosan to alginate is completely covered with curcumin.

The results indicate a clear difference in release rates between curcumin incorporated as a complex and in its pure form. When curcumin is incorporated in a form of inclusion complex, the release rates are significantly higher for all gel compositions; see Table 2. The release profiles, described by the equations w = 11.967 + 0.154 × t (1:4), w = 1.966 + 0.0586 × t (1:1), and w = 1.551 + 0.0269 × t (4:1), revealed a notable trend: a higher proportion of chitosan (1:4) led to the fastest release rate (154 µg/h/g of gel), while a higher proportion of alginate (4:1) resulted in the slowest (26.9 µg/h/g of gel). This trend suggests that the interaction between the complex and the matrix materials (predominantly alginate) significantly affects the release rate.

The results of the release kinetics analysis of curcumin and the curcumin complex from Alg-Chi hydrogels using the described Higuchi and Korsmeyer–Peppas models, are presented in Table 3.

The highest coefficient of determination (R^2^) indicates that the Korsmeyer–Peppas model best describes the release of curcumin from the Alg-Chi hydrogels, whether containing curcumin alone or the curcumin:2-hydroxypropyl-β-cyclodextrin complex. The diffusion exponent values for all samples were below 0.45, suggesting that the release mechanism of both curcumin and its complex is primarily governed by diffusion from the hydrogel matrix. Only for the 4A_1CH_Cu sample was n 0.549, suggesting non-Fickian transport, likely due to the rapid washing off of curcumin from the gel surface, which is evident in the release profile; see Figure 11.

This confirms that the preparation method used produces hydrogels suitable for the sustained release of both pure curcumin and the curcumin complex. The curcumin:2-hydroxypropyl-β-cyclodextrin complex was incorporated to increase the amount of curcumin within the gel. Considering the overall results, it can be concluded that a system for the sustained release of curcumin from a hydrogel-based formulation has been successfully developed. This outcome was primarily achieved by enhancing solubility through the inclusion of curcumin in the 2-hydroxypropyl-β-cyclodextrin complex, which allowed the required amount of curcumin to be incorporated into the hydrogel. Incorporating pure curcumin alone would lead to the formation of curcumin agglomerates within the formulation, whereas the cyclodextrin complex facilitated the diffusion of individual curcumin molecules throughout the gel, significantly enhancing curcumin solubility.

These findings suggest that the interaction between the curcumin complex and the hydrogel components plays a crucial role in controlling release kinetics. This study provides valuable insights for tailoring the release of curcumin or similar hydrophobic drugs complexed with cyclodextrins from alginate-chitosan hydrogels by adjusting the polymer ratio to achieve desired therapeutic outcomes.

## 3. Conclusions

In this work, sodium alginate-chitosan hydrogels were synthesized using microwave-assisted synthesis. This method represents an environmentally friendly approach that aligns with green chemistry principles and sustainability goals. The presence of characteristic peaks for both alginate and chitosan in the FTIR spectra of synthesized hydrogels confirms their successful incorporation. These spectra demonstrate the formation of physically cross-linked networks through intermolecular interactions, primarily hydrogen bonding, between the two polymers. Variations in peak intensity suggest the influence of polymer composition on the extent of hydrogen bonding within the network. The FTIR spectra indicate a higher incorporation or stronger interaction of curcumin in the chitosan-rich hydrogel and a higher incorporation of the curcumin-HPβCD complex in the alginate-rich hydrogel. The glass transition temperature (T_g_) of the hydrogels increased with a higher proportion of chitosan due to chitosan’s greater rigidity and extensive hydrogen bonding interactions, which restrict chain mobility. Conversely, higher chitosan content decreased T_m_ due to the disruption of alginate’s crystalline domains. By incorporating a complex of curcumin and 2-hydroxypropyl-β-cyclodextrin, the solubility and bioavailability of curcumin were significantly enhanced, leading to efficient encapsulation and improved drug delivery performance. Overall, the findings highlight the importance of both the form of curcumin incorporation and the composition of the gel matrix in determining the release profile of curcumin. The chitosan-dominant hydrogel (1:4) exhibited the slowest release rate, while the alginate-dominant hydrogel (4:1) exhibited the fastest. This suggests a stronger interaction between curcumin and chitosan, resulting in a slower release rate of curcumin from gels with a higher amount of chitosan. When curcumin is incorporated in the form of an inclusion complex, a higher proportion of chitosan (1:4) led to the fastest release rate (154 µg/h/g of gel), while a higher proportion of alginate (4:1) resulted in the slowest (26.9 µg/h/g of gel). This trend suggests that the interaction between the complex and the matrix materials (predominantly alginate) significantly affects the release rate. This knowledge can guide the design of curcumin delivery systems for various applications. The successful development of these fully biodegradable, multifunctional hydrogels represents a significant advancement in drug delivery technology. The innovative approach and promising results contribute to ongoing efforts to create more effective, biocompatible, and environmentally sustainable drug delivery systems.

In this study, we successfully synthesized sodium alginate-chitosan hydrogels using a microwave-assisted method, demonstrating a sustainable and environmentally friendly approach in line with green chemistry principles. FTIR analysis confirmed the incorporation of both alginate and chitosan and highlighted the formation of physically cross-linked networks via intermolecular hydrogen bonding. The study revealed that polymer composition significantly influences the extent of hydrogen bonding, thermal properties, and curcumin release profiles within the hydrogels. Notably, the inclusion of curcumin, both as a pure and in complex form with 2-hydroxypropyl-β-cyclodextrin, resulted in varying solubility, bioavailability, and release rates, depending on the alginate-to-chitosan ratio. These results underscore the importance of gel matrix composition in designing effective drug delivery systems, particularly in controlling the release rate of therapeutic agents like curcumin. Although further in vitro and in vivo research as well as toxicity assessments are necessary to fully validate these hydrogels for biomedical applications, our study demonstrates their significant potential. 

## 4. Materials and Methods

### 4.1. Materials

Sodium alginate and chitosan (molecular weight 100,000–300,000 g/mol) were purchased from Acros Organics (Fair Lawn, NJ, USA); Curcumin (Cu), 97%; 2-hydroxypropyl-β-cyclodextrin, 97% (Tokyo Chemical Industry Co., Ltd., Tokyo, Japan); Tween 20 (Alfa Aesar, ThermoFisher, Kandel, Germany); methanol for HPLC, ≥99.9% (Merck KGaA, Darmstadt, Germany); Hanks buffered solution pH 7.4 GmbH (PAA Laboratories, Pasching, Austria).

### 4.2. Synthesis of Hydrogels

For the synthesis of Alg-Chi hydrogels, solutions of sodium alginate and chitosan were first prepared. A 2 wt% solution of sodium alginate in distilled water was made, and the mixture was stirred with a magnetic stirrer until complete dissolution. The pH of this solution was then adjusted to 6. Meanwhile, chitosan was dissolved in 1% acetic acid to obtain a 2 wt% solution, and the pH was adjusted to 4.6 by adding NaOH solution dropwise. Specific volumes of the prepared solutions were measured to achieve Alg-Chi mass ratios of 4:1, 2:1, 1:1, 1:2, and 1:4, with the total sample mass being 4 g; see Table 4. After homogenization with a magnetic stirrer, the samples were placed in a microwave reactor. Polymerization was performed in a “Discover” microwave reactor (CEM Corporation, Matthews, NC, USA) at a frequency of 2.45 GHz and a power of 150 W for 2 min and 30 s. This accelerates the cross-linking process through enhanced ionic interactions and hydrogen bonding. As pointed out by Li and Mooney [38], this approach allows greater control over hydrogel properties by optimizing process parameters such as microwave power and exposure time. Alginate-chitosan hydrogels are formed through physical cross-linking, driven by ionic interactions between the negatively charged carboxyl groups of alginate and the positively charged amine groups of chitosan, with additional stabilization provided by hydrogen bonding [39,40].

### 4.3. Obtaining of the Curcumin:2-hydroxypropyl-β-cyclodextrin Complex

Preparation of the curcumin:2-hydroxypropyl-β-cyclodextrin complex is described in detail in the work of Nikolic et al. [20]. Briefly, 368.38 mg of curcumin was dissolved in 200 cm^3^ of absolute ethanol and added to 100 cm^3^ of 2-hydroxypropyl-β-cyclodextrin solution. The mixture was stirred with a magnetic stirrer at room temperature for 96 h, protected from light. The solution was concentrated on a vacuum evaporator at 40 °C to the minimum volume and then dried in a desiccator to a constant weight. The molar ratio of curcumin and 2-hydroxypropyl-β-cyclodextrin in the inclusion complex was 1:1.

### 4.4. Incorporation of Curcumin and Curcumin:2-hydroxypropyl-β-cyclodextrin Complex

Incorporation of curcumin and curcumin: 2-hydroxypropyl-β-cyclodextrin complex into Alg-Chi gels was performed as follows: a measured mass of xerogels (about 50 mg) was immersed in a solution of curcumin or curcumin:2-hydroxypropyl-β-cyclodextrin complex in ethanol, in which the curcumin concentration was 1.23 mg/cm^3^. The samples were allowed to swell until equilibrium was reached at room temperature, protected from light. After that, the remaining solution of the complex was decanted, and the hydrogels were washed with distilled water. The amount of incorporated curcumin in Alg-Chi hydrogels was determined based on the difference in the amounts of curcumin in the initial curcumin:2-hydroxypropyl-β-cyclodextrin inclusion complex solution and the supernatant after reaching equilibrium using the High-Pressure Liquid Chromatography (HPLC) method. The efficiency of incorporation of curcumin into the hydrogel, η, was calculated according to Equation (1):

(1) $$\eta \;\left(\%\right)=\frac{L_{g}}{L_{u}}\times 100,$$

where L_g_ is mass of curcumin incorporated in the Alg-Chi hydrogel, mg/g_xerogel_, and L_u_ is initial mass of curcumin in the solution of the curcumin:2-hydroxypropylβ-cyclodextrin complex for swelling, mg/g_xerogel_.

### 4.5. Fourier Transform Infrared Spectroscopy (FTIR)

To investigate the chemical structure of curcumin, 2-hydroxypropyl-β-cyclodextrin, the inclusion complex of curcumin:2-hydroxypropyl-β-cyclodextrin, the synthesized xerogels, and the xerogels with an incorporated complex of curcumin: 2-hydroxypropyl-β-cyclodextrin, FTIR spectra were recorded using the technique of thin transparent tablets with potassium bromide of spectroscopic purity (KBr, 99%, Merck, Darmstadt, Germany). Tablets are prepared from about 150mg of KBr, and a certain mass of the sample which was pulverized on an amalgamator is then vacuumed and pressed under a pressure of 200 MPa. FTIR spectra were recorded in the wavelength range numbers from 4000 to 400 cm^−1^ on a Bohm Hartmann & Braun MB-series FTIR spectrophotometer (Hartmann & Braun, Baptiste, Quebec, QC, Canada).

### 4.6. Differential Scanning Calorimetry

Thermal properties of synthesized hydrogels were analyzed by differential scanning calorimetry on the Q20 apparatus (TA Instruments, New Castle, DE, USA). The flow of inert gas (nitrogen) during the test was 50 cm^3^/min. The measurement was performed in the temperature range from −50 to 250 °C at a heating rate of 10 °C/min. A standard calibration of the instrument was performed using indium (T_m_ = 156.6 °C).

### 4.7. Scanning Electron Microscopy (SEM)

Morphology of the xerogels and xerogels with incorporated curcumin or complex curcumin:2-hydroxypropyl-β-cyclodextrin were investigated using a JSM-6460 (JEOL, Tokyo, Japan) scanning electron microscope (SEM) with accelerating voltage of 20 kV. Before recording, the samples were cut and covered with gold on a SCD-005 (Bal-tec/Leica, Wetzlar, Germany) device and investigated at different magnifications.

### 4.8. Swelling Properties of Hydrogels

The degree of swelling of the synthesized hydrogels was determined gravimetrically at different pH values: 3.7, 6.7, and 7.4. The mass of the xerogel was measured, which was then immersed in a solution of the exact pH value, and the mass of the swollen hydrogel was measured at certain time intervals until equilibrium was reached. Equation (2) was used to calculate the degree of swelling (DS):

(2) $$\mathrm{DS}\;(\%)=\frac{m_{1}-m_{0}}{m_{0}}\times 100,$$

where: *m*_1_ is a mass of hydrogel, and *m*_0_ is a mass of xerogel.

### 4.9. Release of Curcumin from Hydrogels

An in vitro study of curcumin release from Alg-Chi hydrogels loaded with the inclusion complex of curcumin:2-hydroxypropyl-β-cyclodextrin and pure curcumin was carried out at 37 °C and pH 7.4, which simulate body temperature and pH conditions in the small intestine. Each sample was covered with 10 cm^3^ of a solution made from 9 cm^3^ buffer with a pH value of 7.4 and 1 cm^3^ Tween 20 solution with a concentration of 1.52 mg/cm^3^. For the next 48 h, the samples were thermostated in a water bath at 37 °C with stirring by a magnetic stirrer. The liquid chromatography (UHPLC: ultrahigh performance liquid chromatography) runs were carried out using a Dionex Ultimate 3000 UHPLC+ system equipped with a diode array detector (DAD), Thermo Fisher Scientific, Waltham, MA 02451, USA. The chromatography was performed on a Hypersil gold C18 column (50 × 2.1 mm, 1.9 μm) from the same producer at 25 °C in an isocratic elution regime. The mobile phase at 0.25 mL/min flow rate and 5 min duration time consisted of 0.1% *v*/*v* formic acid in water and 0.1% *v*/*v* formic acid in methanol in a 5:95 *v*/*v* ratio. The quantification of curcumin was determined by using the external standard method, and the corresponding data (the peak areas of the curcumin) were obtained from chromatograms set at a 425 nm detection wavelength. Xcalibur software (version 2.1) was used for instrument control, data acquisition, and data analysis. Origin 6.0 software was used for data processing.

The first mathematical model designed to describe drug release from a matrix system was introduced by Higuchi in 1961 [41]. Initially developed for planar systems, it was later adapted to various geometries and porous systems, such as hydrogels. In general, the Higuchi model can be simplified and is commonly referred to as the simplified Higuchi model:

f_t_ = Q = K_H_ × t^1/2^ (3)

where K_H_ represents the Higuchi dissolution constant. The data were typically plotted as cumulative percentage drug release versus the square root of time.

Korsmeyer et al. proposed a straightforward equation to describe drug release from a polymeric system [42]:

Mt/M∞ = K_KP_t^n^ (4)

where Mt/M∞ is the fraction of drug released at time t, K is the release rate constant, and n is the release exponent. The value of n is used to characterize different release mechanisms from matrices. The value 0.45 ≤ n indicates the Fickian diffusion; 0.45 < n < 0.890 suggests non-Fickian transport; n = 0.89 corresponds to Case II (relaxational) transport; and n > 0.89 indicates super Case II transport. To determine the exponent n and constant K_KP_, the data for ln(Mt\M∞) should be plotted versus the ln(t).

## Figures and Tables

**Table 1:** Maximum degree of swelling (%) at different pH values.

Sample	pH 7.4	pH 6.7	pH 3.7
1A_4CH	254 ± 7	267 ± 9	284 ± 12
1A_2CH	295 ± 9	297 ± 7	295 ± 10
1A_1CH	280 ± 11	277 ± 13	285 ± 10
2A_1CH	322 ± 15	312 ± 9	325 ± 10
4A_1CH	338 ± 10	328 ± 9	337 ± 12

**Table 2:** The efficiency of curcumin incorporation (η) and release rate from Alg-Chi hydrogels.

Sample	η, (%)	Curcumin Release Rate (µg/h g_gel_)
1A_4CH_Cu	47.9	0.25
1A_4CH_Com	76.6	154
1A_1CH_Cu	35.9	0.245
1A_1CH_Com	87.7	58.6
4A_1CH_Cu	29.7	1.363
4A_1CH_Com	83.6	26.9

**Table 3:** The kinetic of curcumin and curcumin complex release from Alg-Chi hydrogels.

Kinetic Model					
Higuchi $F=k_{H}\cdot t^{1/2}$			Korsmeyer–Peppas $F=k_{KP}\cdot t^{n}$		
Sample	k_H_	R^2^	k_KP_	n	R^2^
1A_4CH_Cu	6.555	−0.394	48.7	0.11685	0.9891
1A_4CH_Com	3.65	−1.114	30.887	0.11658	0.989
1A_1CH_Cu	6.085	−0.669	36.91	0.106	0.990
1A_1CH_Com	4.57	−1.101	42.83	0.226	0.995
4A_1CH_Cu	1.786	−0.283	11.09	0.549	0.9675
4A_1CH_Com	9.4	−0.373	50.01	0.1742	0.9799

**Table 4:** Formulation of prepared Alg-Chi hydrogels with incorporated curcumin and curcumin:2-hydroxypropyl-β-cyclodextrin complex.

Sample	Na-Alginate 2%, g	Chitosan 2%, g	Curcumin, mg	Cu from Cu:HPβCD Complex, mg
1A_4CH	0.8	3.2	/	/
1A_4CH_Cu	0.8	3.2	3.5928	/
1A_4CH_Com	0.8	3.2	/	4.50035
1A_2CH	1.3	2.7	/	/
1A_1CH	2	2	/	/
1A_1CH_Cu	2	2	2.6948	/
1A_1CH_Com	2	2	/	5.15125
2A_1CH	2.7	1.3	/	/
4A_1CH	3.2	0.8	/	/
4A_1CH_Cu	3.2	0.8	2.22725	/
4A_1CH_Com	3.2	0.8	/	4.91075

## Data Availability

The original contributions presented in the study are included in the article, and further inquiries can be directed to the corresponding authors.
